# Cigarette Smoking Is Related to Endothelial Dysfunction of Resistance, but Not Conduit Arteries in the General Population—Results From the Gutenberg Health Study

**DOI:** 10.3389/fcvm.2021.674622

**Published:** 2021-05-19

**Authors:** Omar Hahad, Natalie Arnold, Jürgen H. Prochaska, Marina Panova-Noeva, Andreas Schulz, Karl J. Lackner, Norbert Pfeiffer, Irene Schmidtmann, Matthias Michal, Manfred Beutel, Philipp S. Wild, John F. Keaney, Andreas Daiber, Thomas Münzel

**Affiliations:** ^1^Department of Cardiology—Cardiology I, University Medical Center of the Johannes Gutenberg-University Mainz, Mainz, Germany; ^2^German Center for Cardiovascular Research (DZHK), Partner Site Rhine-Main, Mainz, Germany; ^3^Preventive Cardiology and Preventive Medicine, Department of Cardiology, University Medical Center of the Johannes Gutenberg-University Mainz, Mainz, Germany; ^4^Center for Thrombosis and Hemostasis, University Medical Center of the Johannes Gutenberg-University Mainz, Mainz, Germany; ^5^Institute of Clinical Chemistry and Laboratory Medicine, University Medical Center of the Johannes Gutenberg-University Mainz, Mainz, Germany; ^6^Department of Ophthalmology, University Medical Center of the Johannes Gutenberg-University Mainz, Mainz, Germany; ^7^Institute of Medical Biostatistics, Epidemiology & Informatics, University Medical Center of the Johannes Gutenberg-University Mainz, Mainz, Germany; ^8^Department of Psychosomatic Medicine and Psychotherapy, University Medical Center of the Johannes Gutenberg-University Mainz, Mainz, Germany; ^9^Division of Cardiovascular Medicine, University of Massachusetts Medical School, Worcester, MA, United States

**Keywords:** smoking, endothelial (dys)function, peripheral arterial tonometry, flow-mediated dilation, population-based

## Abstract

**Aims:** Cigarette smoking is one of the most complex and least understood cardiovascular risk factors. Importantly, differences in the tobacco-related pathophysiology of endothelial dysfunction, an early event in atherogenesis, between circulatory beds remain elusive. Therefore, this study evaluated how smoking impacts endothelial function of conduit and resistance arteries in a large population-based cohort.

**Methods and results:** 15,010 participants (aged 35–74 years) of the Gutenberg Health Study were examined at baseline from 2007 to 2012. Smoking status, pack-years of smoking, and years since quitting smoking were assessed by a computer-assisted interview. Endothelial function of conduit and resistance arteries was determined by flow-mediated dilation (FMD) of the brachial artery, reactive hyperemia index (RHI) using peripheral arterial tonometry, as well as by reflection index (RI) derived from digital photoplethysmography, respectively. Among all subjects, 45.8% had never smoked, 34.7% were former smokers, and 19.4% were current smokers. Mean cumulative smoking exposure was 22.1 ± 18.1 pack-years in current smokers and mean years since quitting was 18.9 ± 12.7 in former smokers. In multivariable linear regression models adjusted for typical confounders, smoking status, pack-years of smoking, and years since quitting smoking were independently associated with RHI and RI, while no association was found for FMD. Overall, no clear dose-dependent associations were observed between variables, whereby higher exposure tended to be associated with pronounced resistance artery endothelial dysfunction.

**Conclusions:** Cigarette smoking is associated with altered endothelial function of resistance, but not conduit arteries. The present results suggest that smoking-induced endothelial dysfunction in different circulatory beds may exhibit a differential picture.

## Introduction

In 2015, smoking was ranked second as a leading cause of premature death and disability worldwide (1). According to the report from the World Health Organization on the global tobacco epidemic, an estimated 7 million deaths each year are attributable to tobacco-related diseases (2). The life expectancy of smokers is 20 years less compared with non-smokers, which also explains the devasting socioeconomic burden of 6.6 billion USD of lost productivity (3, 4). Smoking also directly affects the health of others (mostly children) via the harmful effects of second-hand smoke (5). Cigarette smoking is a major reversible risk factor for development and progression of cardiovascular disease (CVD) and ranks among the leading causes of coronary artery disease, ischemic stroke, and peripheral artery disease (6). Even worse, smoking can exert additive adverse health effects with other life style drugs such as alcohol (7, 8) and probably also with common environmental stressors such as traffic noise or air pollution (9).

Smoking contributes to cardiovascular morbidity and mortality through multiple interdependent pathophysiological mechanisms including hemodynamic and autonomic alterations, oxidative stress, inflammation, endothelial dysfunction, thrombosis, and hyperlipidemia (10, 11). Of note, there is a clear dose–response relationship, which means the more you smoke the greater the vascular damage (12). Importantly, altered endothelial function is considered an early key event in smoking-induced atherogenesis. Increasing evidence from clinical and animal studies suggests that exposure to cigarette smoke and its constituents lead to a pathological state of the vascular endothelium initiated by reduced nitric oxide (NO) bioavailability. Altered biosynthesis and decreased activity of NO induced by the reaction of NO with free radicals contained in smoke, as well as the direct physical damage to endothelial cells may subsequently lead to impaired function of the endothelium to maintain its vasodilatory, antithrombotic, anti-inflammatory, and antioxidant effects. These conditions promote atherosclerotic plaque formation, stiffening of the arterial wall, and thus contribute to the pathogenesis of CVD (10, 11, 13).

However, although the adverse effects of smoking as well as the beneficial effects of smoking cessation on cardiovascular morbidity and mortality are well-established (14, 15), little is known about the impact of cigarette smoking and smoking cessation on endothelial function and particularly in different vascular beds. To our knowledge, no prior studies have examined specifically the association between different measures of smoking exposure and cessation and a panel of simultaneously assessed markers of endothelial function in a large population-based cohort that allow differentiating between endothelial function of conduit and resistance arteries. Thus, we sought to evaluate these associations based on data of the population-based Gutenberg Health Study (GHS).

## Methods

### Study Design and Sample

We followed the methods of Hahad et al. (16). In brief, the GHS is a population-based, prospective single-center cohort study from Mid-Western Germany including 15,010 subjects (aged 35–74 years) of the baseline examination performed from April 2007 to April 2012 at the University Medical Center Mainz, Germany. The primary aim of the GHS is to improve cardiovascular risk stratification along with the identification of determinants of metabolic, ophthalmological, cancer, immune system, and mental diseases. After obtaining written informed consent, all participants were assessed on a battery of standardized tests with comprehensive examination of laboratory, lifestyle, psychosocial, and environmental parameters. All procedures conducted in this study were approved by the ethics committee of the Statutory Physician Board of the State Rhineland-Palatinate [reference number 837.020.07(5555)] and the local data safety commissioners. The rationale of the GHS has been described previously (17).

### Ascertainment of Cigarette Smoke Exposure

Smoking information was collected by a computer-assisted interview, including smoking status, pack-years of smoking, and years since quitting smoking. According to the smoking status, subjects were classified as never, former, or current smokers. Current smoking was defined as regular or daily smoking (at least 1 cigarette per day, 7 per week, or 1 pack per month) for at least the past 6 months. Never smoking was defined as never having smoked daily or regularly and those who quit smoking were defined as former smokers. Current and former smokers were asked for year of smoking initiation/cessation and average consumption of cigarettes per day. To estimate cumulative smoking exposure in current smokers, pack-years of smoking were calculated as number of cigarettes smoked per day divided by 20 (a pack) and multiplied by the number of years smoked. Passive smoking among never and former smokers was defined as being exposed to cigarette smoke at home, workplace, and/or elsewhere (e.g., bars, clubs, restaurants) for at least half an hour per day. Participants were advised not to smoke for at least 6 h prior to examination in order to avoid acute effects of smoking.

### Assessment of Endothelial Function

Flow-mediated dilation (FMD) of the brachial artery was measured to determine conduit artery endothelial function. Under standardized conditions, FMD was measured after a 5-min upper-arm occlusion as percentage increase of brachial artery diameter in resting conditions. Two-dimensional high-resolution ultrasound images of the right brachial artery were acquired with a Philips HD11XE CV ultrasound machine (Best, The Netherlands) using a linear array broadband probe, L12–5 (38 mm). Artery diameters were analyzed offline with Brachial Analyzer software tool (version 5.0, Medical Imaging Applications LLC; Iowa City, IA).

For digital peripheral arterial tonometry, Endo-PAT2000 device (Itamar Medical, Caesarea, Israel) was used to estimate resistance artery endothelial function by recording digital pulsatile volume changes. Reactive hyperemia index (RHI) was calculated as logarithmic ratio between rest and post-occlusion in digital pulse amplitude, normalized to the left control finger.

Digital pulse waveform volume was registered electronically by measuring the absorption of infrared light at 940 nm through the finger pulp with a PulseTrace 2000 device (Micro Medical Limited/Carefusion) and pulse waveform was automatically analyzed. The plethysmography transducer, a non-invasive finger clip, was placed on the subject's ring (or fourth) finger and 10 pulses were recorded to produce a representative pulse waveform. This waveform consists of an early systolic and a second diastolic peak. Reflection index (RI) was defined as the ratio amplitude of the forward wave (early systolic) and reflected wave (second diastolic peak) component.

All measurements were performed simultaneously in a single examination according to standard operating procedures by trained technicians with an experience of at least 250 vascular function studies before study enrollment and with continuing quality assessment. Reproducibility of the measurements was evaluated and provided good intraclass and interclass variability for all measures of endothelial function (e.g., for FMD: 0.87–0.93 and 0.90–0.93, respectively). Further details and quality control data about the good reproducibility of the different endothelial function measurement methods in the GHS have been described previously (18, 19).

### Measurement of Cardiovascular Risk Factors, Diseases, and Psychosocial Variables

Participants underwent standardized interviews on cardiovascular risk factors, CVD, lifestyle, and psychosocial variables. Medication intake was obtained by self-report and participants were advised to bring their current medications. Prevalent CVD was recorded by self-report or diagnosed during study visit. Fasting blood and anthropometric data were collected and routine laboratory methods were used for measurements of blood glucose, lipids, and cardiovascular risk factors. Detailed information about clinical and laboratory examinations as well as exact definition of included variables have been reported previously (16, 20, 21).

### Statistical Analysis

Study sample characteristics are presented according to smoking status (i.e., never, former, current). We used linear regression analysis to determine the association between smoking status, pack-years of smoking, and years since quitting smoking with parameters of endothelial function. To further elucidate the association between pack-years of smoking, years since quitting smoking, and the outcomes, exposure variables were modeled as categories into linear regression analysis and effect plots were generated. Separate analyses for each marker of endothelial function were performed. The basic model was adjusted for sex (male or female) and age (continuous); the comprehensive model was further adjusted for arterial hypertension (yes or no), diabetes mellitus (yes or no), waist-to-height ratio (continuous), dyslipidemia (yes or no), family history of myocardial infarction or stroke (yes or no), socioeconomic status (continuous), alcohol consumption above tolerable limit (yes or no), depression (yes or no), physical activity (continuous), passive smoking (yes or no), smoking prior to examination (yes or no), prevalent CVD (yes or no; composite variable comprising coronary artery disease, peripheral artery disease, myocardial infarction, congestive heart failure, stroke, and atrial fibrillation), and medication use (yes or no for diabetic drugs, antithrombotic agents, antihypertensives, diuretics, beta-blockers, calcium channel blocker, agents acting on the renin-angiotensin-aldosterone system, and lipid modifying agents). All tests were two-sided with a significance level of 5%. Since these analyses are of exploratory nature, no adjustments of *p*-values for multiple testing were done. The software R, version 3.6.0 (http://www.r-project.org/) was used to perform the statistical data analyses.

## Results

### Characteristics of the Study Sample

Of the study sample with 15,010 subjects initially enrolled, 14,975 (99.8%) provided information on smoking status. Of those, 6,863 (45.8%) were never, 5,201 (34.7%) were former, and 2,911 (19.4%) were current smokers. Characteristics of the study sample according to smoking status are described in Table 1. Individuals with a positive history of smoking (i.e., former and current smokers) were more likely to be male, had a lower socioeconomic status, and higher prevalence of depression as well as alcohol consumption above tolerable limit compared to never smokers. In general, current smokers were 4 or 5 years younger than never and former smokers. Pack-years of smoking were greater in current than in former smokers. Exposure to passive smoking has been found to be higher in former than never smokers. More than half of current smokers smoked at least 6 h prior to examination. Concerning traditional cardiovascular risk factors, former smokers demonstrated the highest prevalence of arterial hypertension, diabetes mellitus, dyslipidemia, and a positive family history of myocardial infarction or stroke. A similar trend was observed for manifest CVD, where prevalences were higher in former than in never and current smokers. Also, the use of cardiac medication was highest among former smokers. With regard to endothelial function markers, former and current smokers had reduced FMD, lower RHI, and increased RI, compared to never smokers.

**Table 1:** Characteristics of the study sample by smoking status (*N* = 14,975)^*^.

	**Never**	**Former**	**Current**
	**(*n* = 6,863)**	**(*n* = 5,201)**	**(*n* = 2,911)**
**Characteristic**			
Female sex – no. (%)	3,960 (57.7)	2,116 (40.7)	1,335 (45.9)
Age—years	55.3 ± 11.8	56.7 ± 10.4	51.3 ± 9.7
Physical activity^†^	7.29 ± 3.79	7.15 ± 3.98	7.92 ± 4.41
Waist-to-height ratio^‡^	0.55 ± 0.08	0.57 ± 0.08	0.55 ± 0.08
Alcohol consumption above tolerable limit—no. (%)^§^	1,209 (17.6)	1,415 (27.2)	740 (25.4)
Socioeconomic status^||^	13.06 ± 4.64	13.02 ± 4.37	12.25 ± 4.22
Depression – no. (%)^#^	466 (6.9)	352 (6.9)	314 (11.0)
**Smoking**			
Pack-years	–	1.57 (0.57/3.54)	18.70 (8.40/31.79)
Years since quitting	–	18.0 (8.00/29.00)	–
Passive smoking – no. (%)	934 (13.6)	952 (18.3)	–
Smoked < 6 h prior to examination – no. (%)	–	–	1,646 (56.5)
**Traditional cardiovascular risk factors – no. (%)**			
Arterial hypertension	3,424 (49.9)	2,878 (55.4)	1,147 (39.4)
Diabetes mellitus	558 (8.2)	601 (11.6)	228 (7.8)
Dyslipidemia	2,136 (31.2)	2,019 (38.9)	1,009 (34.7)
Family history of myocardial infarction or stroke	1,435 (20.9)	1,211 (23.3)	670 (23.0)
**Cardiovascular comorbidities – no. (%)**			
Congestive heart failure	476 (6.9)	430 (8.3)	242 (8.3)
Coronary artery disease	217 (3.2)	329 (6.4)	94 (3.3)
Myocardial infarction	123 (1.8)	234 (4.5)	85 (2.9)
Stroke	104 (1.5)	128 (2.5)	47 (1.6)
Atrial fibrillation	1,169 (17.0)	1,003 (19.3)	527 (18.1)
Peripheral artery disease	189 (2.8)	207 (4.0)	106 (3.7)
Any cardiovascular disease	1,853 (27.2)	1,698 (32.9)	852 (29.4)
**Measurements of endothelial function**			
Flow-mediated dilation – %	8.41 ± 5.41	7.70 ± 4.91	8.36 ± 5.37
Baseline brachial artery diameter – mm	4.24 ± 0.84	4.47 ± 0.87	4.26 ± 0.82
Reactive hyperemia index	0.69 ± 0.41	0.61 ± 0.41	0.62 ± 0.42
Baseline pulse amplitude – mm	389.4 (192.6/754.5)	513.3 (242.7/896.4)	469.8 (230.9/839.4)
Reflection index	63.42 ± 16.33	66.24 ± 15.80	67.92 ± 15.37
Medication – no. (%)^**^			
Diabetic drugs (A10)	382 (5.6)	406 (7.9)	130 (4.5)
Antithrombotic agents (B01)	738 (10.9)	827 (16.0)	274 (9.5)
Antihypertensives (C02)	74 (1.1)	63 (1.2)	18 (0.6)
Diuretics (C03)	359 (5.3)	318 (6.2)	109 (3.8)
Beta-blockers (C07)	1,148 (16.9)	1,011 (19.6)	370 (12.9)
Calcium channel blocker (C08)	490 (7.2)	437 (8.5)	158 (5.5)
Agents acting on the renin-angiotensin-aldosterone system (C09)	1,545 (22.8)	1,478 (28.7)	503 (17.5)
Lipid modifying agents (C10)	843 (12.4)	877 (17.0)	258 (9.0)

* *Plus-minus values are means ± standard deviation and two values in parentheses are medians with 25 and 75th percentiles.*

† *Physical activity score was calculated by multiplying total minutes of activity by the intensity score displayed per 1,000-units.*

‡ *Waist-to-height ratio is the waist circumference divided by the body height in centimeters.*

§ *Alcohol consumption above tolerable limit denotes >24 g per day for men and >12 g per day for women.*

|| *Socioeconomic status score ranges from 3 to 21 with higher values indicating higher status.*

# *Caseness of depression was indicated by a PHQ-9 score ≥10*.

** *Medication is labeled with the anatomical therapeutic chemical-code.*

### Association Between Smoking Status and Endothelial Function

**Figure 1:**
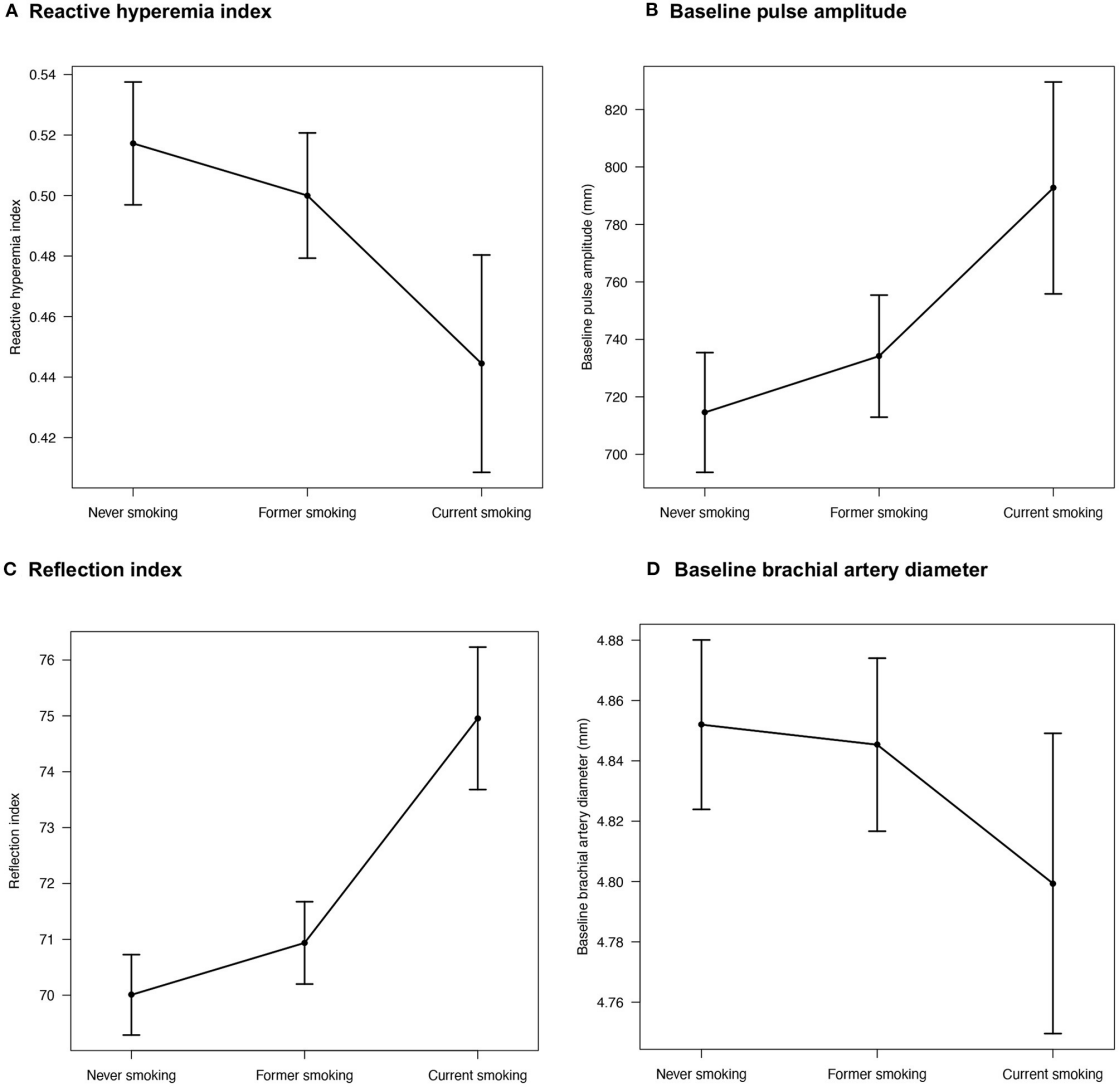
Effect plots demonstrating the relationship between smoking status and endothelial function markers. Adjusted mean values are derived from a linear regression model and beta estimates, 95% confidence intervals, and adjustment are shown in Table 2. Only variables with relevant associations (*p* < 0.05) are displayed. Sample sizes were for **(A)** reactive hyperemia index *N* = 8,690, **(B)** baseline pulse amplitude *N* = 8,690, **(C)** reflection index *N* = 10,691, and **(D)** baseline brachial artery diameter *N* = 10,651.

To quantify the associations between smoking status and several parameters of endothelial function, linear regression modeling with multivariable adjustment was performed (never smoking was used as a reference category) (Table 2) and effect plots with adjusted mean values for relevant associations were generated (Figure 1). Smoking status was independently and inversely associated with RHI after adjustment for potential confounders such as sex, age, arterial hypertension, diabetes mellitus, waist-to-height ratio, dyslipidemia, family history of myocardial infarction or stroke, socioeconomic status, alcohol consumption, depression, physical activity, passive smoking, smoking prior to examination, prevalent CVD, and medication use with higher effect estimates for current than former smoking. Furthermore, baseline pulse amplitude was independently related to former and current smoking in a dose-dependent manner. Likewise, RI was independently associated with former and current smoking. In contrast, no relationship between smoking status and FMD was observed. The baseline brachial artery diameter was independently and inversely associated with current smoking, while no association was observed for former smoking.

**Table 2:** Associations between smoking status and endothelial function markers ^*^.

	**Model 1 ^†^ beta estimate [95% CI]**	***P*-value**	**Model 2 ^‡^ beta estimate [95% CI]**	***P*-value**
**Estimates for flow-mediated dilation**				
Never smoking (ref.)	–	–	–	–
Current smoking	−0.045 [−0.28; 0.19]	0.70	0.10 [−0.26; 0.46]	0.58
Former smoking	−0.010 [−0.20; 0.18]	0.92	0.042 [−0.18; 0.27]	0.71
**Estimates for baseline brachial artery diameter**				
Never smoking (ref.)	–	–	–	–
Current smoking	−0.036 [−0.063; −0.0092]	**0.0084**	−0.046 [−0.087; −0.0049]	**0.028**
Former smoking	0.0071 [−0.015; 0.029]	0.54	−0.0066 [−0.032; 0.019]	0.61
**Estimates for reactive hyperemia index**				
Never smoking (ref.)	–	–	–	–
Current smoking	−0.065 [−0.085; −0.045]	** < 0.0001**	−0.056 [−0.085; −0.026]	**0.00024**
Former smoking	−0.031 [−0.048; −0.015]	**0.00022**	−0.017 [−0.036; 0.0013]	0.069
**Estimates for baseline pulse amplitude**				
Never smoking (ref.)	–	–	–	–
Current smoking	50 [30; 70]	** < 0.0001**	59 [28; 89]	**0.00016**
Former smoking	27 [11; 44]	**0.0014**	20 [0.54; 39]	**0.044**
**Estimates for reflection index**				
Never smoking (ref.)	–	–	–	–
Current smoking	3.5 [2.8; 4.1]	** < 0.0001**	4.0 [3.0; 5.1]	** < 0.0001**
Former smoking	0.79 [0.22; 1.3]	**0.0061**	0.93 [0.27; 1.6]	**0.0055**

* *Beta estimates and 95% confidence intervals are derived from a linear regression model modeling for endothelial function. Current and former smoking were compared to never smoking (reference category). Sample sizes (model 2) were for flow-mediated dilation N = 9,828, baseline brachial artery diameter N = 10,651, reactive hyperemia index N = 8,690, baseline pulse amplitude N = 8,690, and reflection index N = 10,691.*

† *Model 1 was adjusted for sex and age.*

‡ *Model 2 was additionally adjusted for arterial hypertension, waist-to-height ratio, diabetes mellitus, dyslipidemia, family history of myocardial infarction or stroke, socioeconomic status, alcohol consumption, physical activity, depression, passive smoking, smoking prior to examination, prevalent cardiovascular disease (compromising congestive heart failure, coronary artery disease, myocardial infarction, stroke, atrial fibrillation, and peripheral artery disease), and medication use (diabetic drugs, antithrombotic agents, antihypertensives, diuretics, beta-blockers, calcium channel blocker, agents acting on the renin-angiotensin-aldosterone system, and lipid modifying agents).*

*Statistically significant P-values < 0.05 are given in bold.*

### Association Between Pack-Years of Smoking and Endothelial Function

**Figure 2:**
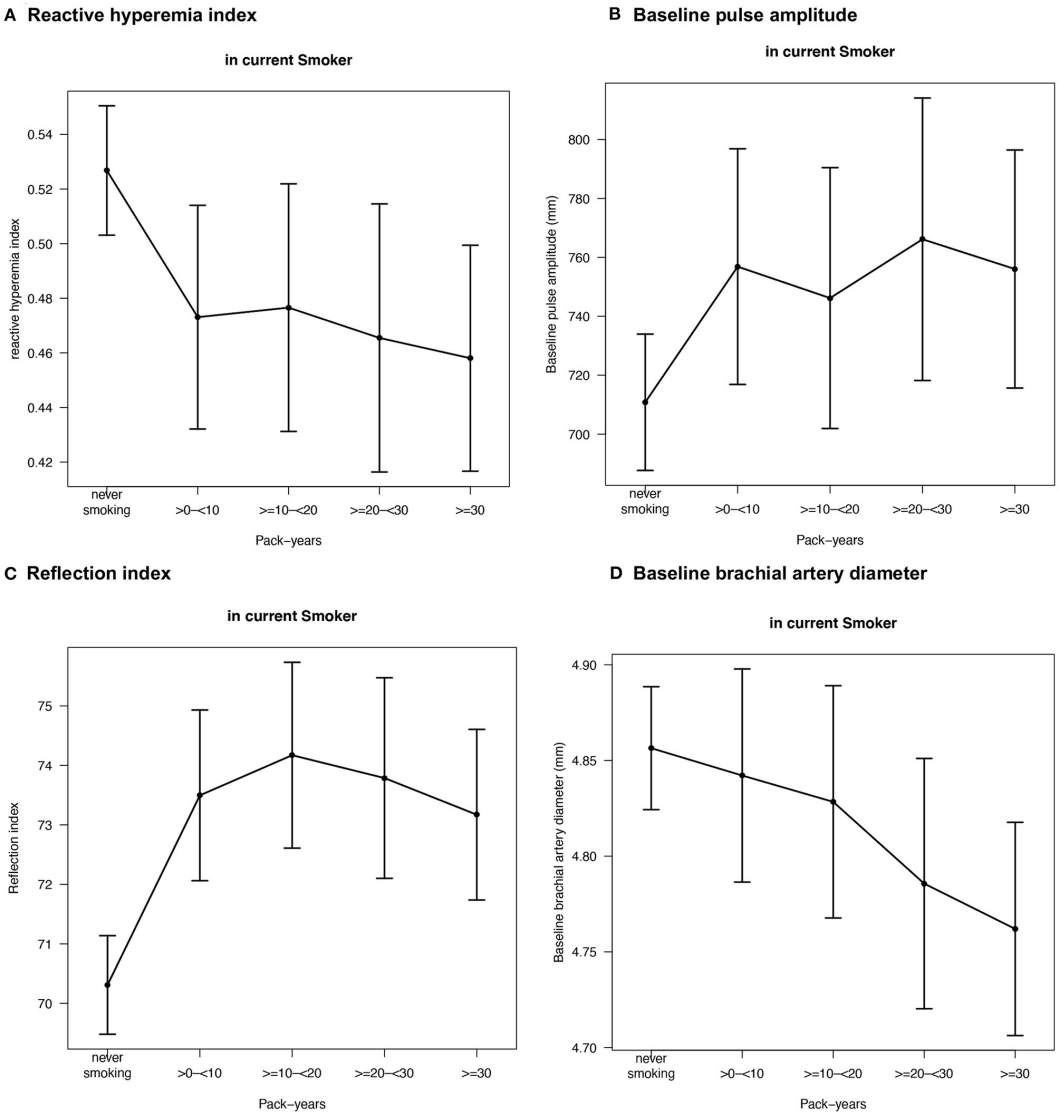
Effect plots demonstrating the relationship between pack-years of smoking in current smokers and endothelial function markers. Adjusted mean values are derived from a linear regression model and beta estimates, 95% confidence intervals, and adjustment are shown in Table 3. Only variables with relevant associations (*p* < 0.05) are displayed. Sample sizes were for **(A)** reactive hyperemia index *N* = 5,424, **(B)** baseline pulse amplitude *N* = 5,424, **(C)** reflection index *N* = 6,706, and **(D)** baseline brachial artery diameter *N* = 6,660.

To further assess the association between cumulative smoking exposure in current smokers and endothelial function markers, pack-years of smoking were modeled as categories of exposure (>0– < 10, ≥10– < 20, ≥20– < 30, ≥30 pack-years; never smoking was used as a reference category) into linear regression analysis (Table 3). Effect plots are presented in the Figure 2. Again, no association was found for FMD across the categories of pack-years of smoking, whereas RI, RHI, and baseline pulse amplitude were consistently associated with categories of smoking exposure. Associations for baseline brachial artery diameter were found to be weaker, while higher exposure categories (i.e., ≥20– < 30 and ≥30 pack-years) were independently and inversely related to baseline brachial artery diameter. The pattern of results found for the impact of heavy smoking (i.e., < 20 vs. ≥20 pack-years) was similar, showing that RI, RHI, and baseline pulse amplitude were consistently associated, whereas no association in case of FMD and a weaker association in case of brachial artery diameter were found (Table 4).

**Table 3:** Associations between pack-years of smoking in current smokers and endothelial function markers ^*^.

	**Model 1 ^†^ beta estimate [95% CI]**	***P*-value**	**Model 2 ^‡^ beta estimate [95% CI]**	***P*-value**
**Pack-years of smoking**	**Estimates for flow-mediated dilation**			
Never smoking (ref.)	–	–	–	–
>0– < 10	0.057 [−0.36; 0.47]	0.79	0.057 [−0.41; 0.52]	0.81
≥10– < 20	−0.24 [−0.69; 0.21]	0.30	−0.24 [−0.75; 0.28]	0.37
≥20– < 30	−0.19 [−0.70; 0.32]	0.46	−0.11 [−0.67; 0.46]	0.71
≥30	0.014 [−0.40; 0.43]	0.95	0.061 [−0.42; 0.54]	0.80
	**Estimates for baseline brachial artery diameter**			
Never smoking (ref.)	–	–	–	–
>0– < 10	−0.0036 [−0.050; 0.043]	0.88	−0.014 [−0.065; 0.036]	0.58
≥10– < 20	−0.035 [−0.085; 0.015]	0.17	−0.028 [−0.084; 0.028]	0.32
≥20– < 30	−0.045 [−0.10; 0.012]	0.12	−0.071 [−0.13; −0.0094]	**0.024**
≥30	−0.060 [−0.11; −0.013]	**0.012**	−0.094 [−0.15; −0.042]	**0.00038**
	**Estimates for reactive hyperemia index**			
Never smoking (ref.)	–	–	–	–
>0– < 10	−0.075 [−0.11; −0.040]	** < 0.0001**	−0.054 [−0.091; −0.017]	**0.0045**
≥10– < 20	−0.038 [−0.076; 0.00017]	0.051	−0.050 [−0.092; −0.0086]	**0.018**
≥20– < 30	−0.060 [−0.10; −0.017]	**0.0063**	−0.061 [−0.11; −0.015]	**0.0092**
≥30	−0.077 [−0.11; −0.041]	** < 0.0001**	−0.069 [−0.11; −0.030]	**0.00050**
	**Estimates for baseline pulse amplitude**			
Never smoking (ref.)	–	–	–	–
>0– < 10	62 [28; 95]	**0.00029**	46 [9.8; 82]	**0.013**
≥10– < 20	26 [−12; 63]	0.18	35 [−5.3; 76]	0.089
≥20– < 30	63 [21; 105]	**0.0031**	55 [10; 100]	**0.016**
≥30	50 [16; 84]	**0.0044**	45 [7.4; 83]	**0.019**
	**Estimates for reflection index**			
Never smoking (ref.)	–	–	–	–
>0– < 10	3.6 [2.4; 4.8]	** < 0.0001**	3.2 [1.9; 4.5]	** < 0.0001**
≥10– < 20	4.5 [3.3; 5.8]	** < 0.0001**	3.9 [2.4; 5.3]	** < 0.0001**
≥20– < 30	3.2 [1.8; 4.7]	** < 0.0001**	3.5 [1.9; 5.1]	** < 0.0001**
≥30	2.8 [1.6; 4.0]	** < 0.0001**	2.9 [1.5; 4.2]	** < 0.0001**

* *Beta estimates and 95% confidence intervals are derived from a linear regression model modeling for endothelial function. Pack-years were modeled as categories (the reference category was never smoking). Sample sizes (model 2) were for flow-mediated dilation N = 6,143, baseline brachial artery diameter N = 6,660, reactive hyperemia index N = 5,424, baseline pulse amplitude N = 5,424, and reflection index N = 6,706.*

† *Model 1 was adjusted for sex and age.*

‡ *Model 2 was additionally adjusted for arterial hypertension, waist-to-height ratio, diabetes mellitus, dyslipidemia, family history of myocardial infarction or stroke, socioeconomic status, alcohol consumption, physical activity, depression, passive smoking, smoking prior to examination, prevalent cardiovascular disease (compromising congestive heart failure, coronary artery disease, myocardial infarction, stroke, atrial fibrillation, and peripheral artery disease), and medication use (diabetic drugs, antithrombotic agents, antihypertensives, diuretics, beta-blockers, calcium channel blocker, agents acting on the renin-angiotensin-aldosterone system, and lipid modifying agents).*

*Statistically significant P-values < 0.05 are given in bold.*

**Table 4:** Associations between heavy smoking and endothelial function markers ^*^.

	**Model 1 ^†^ beta estimate [95% CI]**	***P*-value**	**Model 2 ^‡^ beta estimate [95% CI]**	***P*-value**
**Pack-years of smoking**	**Estimates for flow-mediated dilation**			
Never smoking (ref.)	–	–	–	–
<20	−0.0099 [−0.32; 0.30]	0.95	−0.041 [−0.39; 0.31]	0.82
≥20	−0.067 [−0.39; 0.26]	0.69	−0.060 [−0.43; 0.31]	0.75
	**Estimates for baseline brachial artery diameter**			
Never smoking (ref.)		–	–	–
<20	−0.022 [−0.058; 0.014]	0.23	−0.022 [−0.062; 0.017]	0.27
≥20	−0.057 [−0.094; −0.019]	**0.0029**	−0.083 [−0.12; −0.042]	** < 0.0001**
	**Estimates for reactive hyperemia index**			
Never smoking (ref.)	–	–	–	–
<20	−0.061 [−0.088; −0.035]	** < 0.0001**	−0.054 [−0.082; −0.025]	**0.00022**
≥20	−0.070 [−0.097; −0.042]	** < 0.0001**	−0.063 [−0.094; −0.033]	** < 0.0001**
	**Estimates for baseline pulse amplitude**			
Never smoking (ref.)	–	–	–	–
<20	46 [19; 73]	**0.00071**	43 [14; 72]	**0.0038**
≥20	53 [25; 81]	**0.00021**	49 [18; 80]	**0.0020**
	**Estimates for reflection index**			
Never smoking (ref.)	–	–	–	–
<20	3.9 [3.0; 4.8]	** < 0.0001**	3.4 [2.3; 4.4]	** < 0.0001**
≥20	2.9 [2.0; 3.9]	** < 0.0001**	3.1 [2.0; 4.2]	** < 0.0001**

* *Beta estimates and 95% confidence intervals are derived from a linear regression model modeling for endothelial function. Pack-years were modeled as categories (the reference category was never smoking). Sample sizes (model 2) were for flow-mediated dilation N = 9,608, baseline brachial artery diameter N = 10,416, reactive hyperemia index N = 8,500, baseline pulse amplitude N = 8,500, and reflection index N = 10,459.*

† *Model 1 was adjusted for sex and age.*

‡ *Model 2 was additionally adjusted for arterial hypertension, waist-to-height ratio, diabetes mellitus, dyslipidemia, family history of myocardial infarction or stroke, socioeconomic status, alcohol consumption, physical activity, depression, passive smoking, smoking prior to examination, prevalent cardiovascular disease (compromising congestive heart failure, coronary artery disease, myocardial infarction, stroke, atrial fibrillation, and peripheral artery disease), and medication use (diabetic drugs, antithrombotic agents, antihypertensives, diuretics, beta-blockers, calcium channel blocker, agents acting on the renin-angiotensin-aldosterone system, and lipid modifying agents).*

*Statistically significant P-values < 0.05 are given in bold.*

### Association Between Years Since Quitting Smoking and Endothelial Function

To evaluate the association between years since quitting smoking in former smokers and endothelial function markers, years since quitting smoking were modeled as categories (>0– < 5, ≥5– < 10, ≥10– < 20, ≥20– < 30, ≥30 years; current smoking was the reference category) into linear regression analysis (Table 5). Reflection index was consistently associated with years since quitting smoking across all categories, whereas the associations with RHI, baseline pulse amplitude, and baseline brachial artery diameter were weaker, indicating stronger effects starting at around ≥10– < 20 years after quitting smoking. No association was observed in case of FMD.

**Table 5:** Associations between years since quitting smoking in former smokers and endothelial function markers ^*^.

	**Model 1 ^†^ beta estimate [95% CI]**	***P*-value**	**Model 2 ^‡^ beta estimate [95% CI]**	***P*-value**
**Years since quitting smoking**	**Estimates for flow-mediated dilation**			
Current smoking (ref.)	–	–	–	–
>0– < 5	−0.25 [−0.70; 0.19]	0.26	−0.15 [−0.64; 0.34]	0.55
≥5– < 10	−0.35 [−0.75; 0.056]	0.091	−0.13 [−0.58; 0.33]	0.58
≥10– < 20	0.16 [−0.19; 0.50]	0.37	0.24 [−0.15; 0.62]	0.22
≥20– < 30	−0.23 [−0.59; 0.13]	0.21	−0.14 [−0.54; 0.26]	0.50
≥30	0.11 [−0.26; 0.48]	0.57	0.28 [−0.13; 0.70]	0.19
	**Estimates for baseline brachial artery diameter**			
Current smoking (ref.)	–	–	–	–
>0– < 5	0.061 [0.0079; 0.11]	**0.024**	0.035 [−0.022; 0.092]	0.23
≥5– < 10	0.080 [0.031; 0.13]	**0.0013**	0.053 [−0.00032; 0.11]	0.051
≥10– < 20	0.041 [0.00016; 0.082]	**0.049**	0.033 –[0.012; 0.078]	0.15
≥20– < 30	0.053 [0.010; 0.096]	**0.015**	0.055 [0.0076; 0.10]	**0.023**
≥30	0.061 [0.017; 0.11]	**0.0069**	0.063 [0.014; 0.11]	**0.011**
	**Estimates for reactive hyperemia index**			
Current smoking (ref.)	–	–	–	–
>0– < 5	0.0022 [−0.037; 0.041]	0.91	0.032 [−0.0084; 0.073]	0.12
≥5– < 10	−0.011 [−0.046; 0.025]	0.56	0.0057 [−0.032; 0.043]	0.77
≥10– < 20	0.041 [0.011; 0.071]	**0.0074**	0.044 [0.012; 0.076]	**0.0078**
≥20– < 30	0.043 [0.011; 0.074]	**0.0082**	0.033 [−0.000040; 0.067]	0.053
≥30	0.072 [0.039; 0.10]	** < 0.0001**	0.056 [0.021; 0.091]	**0.0019**
	**Estimates for baseline pulse amplitude**			
Current smoking (ref.)	–	–	–	–
>0– < 5	44 [4.1; 84]	**0.031**	8.0 [−35; 51]	0.72
≥5– < 10	3.0 [−34; 40]	0.87	−18 [−58; 22]	0.38
≥10– < 20	−31 [−62; 0.28]	0.052	−41 [−75; −6.7]	**0.019**
≥20– < 30	−35 [−68; −2.3]	**0.036**	−16 [−52; 20]	0.37
≥30	−42 [−76; −8.2]	**0.015**	−37 [−75; −0.055]	**0.050**
	**Estimates for reflection index**			
Current smoking (ref.)	–	–	–	–
>0– < 5	−2.3 [−3.6; −1.0]	**0.00049**	−2.3 [−3.7; −0.81]	**0.0023**
≥5– < 10	−1.4 [−2.6; −0.19]	**0.023**	−1.7 [−3.0; −0.36]	**0.013**
≥10– < 20	−3.1 [−4.4; −2.4]	** < 0.0001**	−3.1 [−4.2; −2.0]	** < 0.0001**
≥20– < 30	−2.6 [−3.6; −1.5]	** < 0.001**	−2.2 [−3.4; −1.1]	**0.00019**
≥30	−1.9 [−3.0; −0.77]	**0.00083**	−1.9 [−3.1; −0.64]	**0.0029**

* *Beta estimates and 95% confidence intervals are derived from a linear regression model modeling for endothelial function. Years since quitting were modeled as categories (the reference category was current smoking). Sample sizes (model 2) were for flow-mediated dilation N = 5,397, baseline brachial artery diameter N = 5,852, reactive hyperemia index N = 4,776, baseline pulse amplitude N = 4,776, and reflection index N = 5,871.*

† *Model 1 was adjusted for sex and age.*

‡ *Model 2 was additionally adjusted for arterial hypertension, waist-to-height ratio, diabetes mellitus, dyslipidemia, family history of myocardial infarction or stroke, socioeconomic status, alcohol consumption, physical activity, depression, passive smoking, prevalent cardiovascular disease (compromising congestive heart failure, coronary artery disease, myocardial infarction, stroke, atrial fibrillation, and peripheral artery disease), and medication use (diabetic drugs, antithrombotic agents, antihypertensives, diuretics, beta-blockers, calcium channel blocker, agents acting on the renin-angiotensin-aldosterone system, and lipid modifying agents).*

*Statistically significant P-values < 0.05 are given in bold.*

## Discussion

To our knowledge, this is the first study that particularly examined the relationship between measures of cigarette smoking exposure and cessation and a panel of simultaneously assessed markers of endothelial function in a large population-based cohort, allowing differentiation between endothelial function of conduit and resistance arteries. The main findings of the present study are as follows: Firstly, smoking exposure was associated with a worsening and cessation with an improvement of resistance artery endothelial function as indicated by RHI and RI. Secondly, these relationships were not seen for FMD, a marker of conduit artery endothelial function, thus highlighting a differential vascular endothelial profile with regard to exposure to cigarette smoke. Thirdly, adjustment for a broad range of confounders such as sex, age, traditional cardiovascular risk factors, prevalent CVD, and medication use attenuated the effect estimates only slightly, implicating that smoking exposure constitutes a strong independent risk marker for dysregulated endothelial function of resistance arteries.

### Smoking-Induced Endothelial Dysfunction

Endothelial dysfunction represents an early subclinical vascular consequence in the development of atherosclerotic CVD (22). Furthermore, endothelial dysfunction has been shown to have prognostic value for the prediction of cardiovascular events in various disease phenotypes including patients with arterial hypertension, coronary or peripheral artery disease, and chronic congestive heart failure (23). Therefore, its early detection may be useful in the risk stratification for CVD. In the setting of smoking-induced endothelial dysfunction, there is increasing evidence for a role of oxidative stress and inflammation as crucial factors, conferring smoking-dependent endothelial damage and therefore acceleration of vascular aging (24–29). A possible mechanism by which exposure to cigarette smoke and its constituents (mainly toxic species such as free radicals and reactive aldehydes) induces endothelial dysfunction is the reduced NO bioavailability and further the increased expression of adhesion molecules (10). Smoking-induced abnormalities in NO production result in increased adherence of platelets and macrophages to the vessel wall, which provokes the progression of a procoagulant and inflammatory environment (10). Phagocytic NADPH oxidase (NOX-2) was identified as a major contributor to cigarette smoking-induced oxidative stress by cell culture and animal studies (29, 30). Although the relevance of NOX-2 for smoking associated cardiovascular complications in humans is so far not supported by associations of NADPH oxidase centered inactivating polymorphisms, there is clinical evidence that NOX-2 activation plays a pathophysiological role in smoking. Children who are exposed to passive smoke have higher soluble NOX-2-derived peptide (sNOX2-dp, activation marker of NOX-2 enzyme) levels in association with higher oxidative stress marker levels of 8-isoprostane, lower NO bioavailability, and impaired endothelial function (measured by FMD) (31). Additionally, active smokers displayed higher sNOX2-dp levels, more pronounced translocation of the NADPH oxidase subunit p47phox to the membrane of platelets (indicator of NOX-2 activation), decreased nitric oxide metabolite levels, and impaired endothelial function (determined by FMD), all of which was ameliorated by epicatechin, an antioxidant from dark chocolate (32). Mitochondrial oxidative stress was reported as another important contributor to cigarette smoking-induced endothelial dysfunction and hypertension in animals (33). As previously reviewed, the higher content of toxic compounds in water pipe (shisha) smoke suggests even higher levels of oxidative stress in shisha smokers (34), supporting the increasing incidence of CVD, especially among young smokers (29). Of note, the impact of oxidative stress on endothelial dysfunction in smokers was previously demonstrated by several clinical studies (35–37) and also in patients with coronary artery disease (38) [for review see (39)].

A number of non-invasive methods are available for the *in-vivo* assessment of conduit and resistance artery endothelial function (22). In 1993, a landmark study by Celermajer et al. showed that cumulative smoking exposure impairs FMD of the brachial artery in healthy young adults (40). In the following years, evidence for smoking-induced endothelial dysfunction has emerged from an increasing number of studies measuring endothelial function by use of different techniques (41). Flow-mediated dilation of the brachial artery has become the most widely used method for the measurement of endothelial function, while other methods (e.g., peripheral arterial tonometry or digital photoplethysmography) have gained increasing relevance due to advantages such as their simplicity, easy feasibility with low intra- and interobserver variability, as well as a possibility of using the contralateral arm to adjust for systematic drifts (22). However, differences in the pathophysiology of smoking-induced endothelial dysfunction between circulatory beds, i.e., conduit and resistance arteries, remain poorly investigated. The present study clearly showed that in the context of cigarette smoking the assessment of endothelial function in different vascular beds shows a differential picture highlighting the different physiological role of conduit and resistance arteries.

### Smoking-Induced Endothelial Dysfunction of Conduit and Resistance Arteries

Although cardiovascular risk factors have been shown to promote endothelial dysfunction in mostly every arterial bed, the examination of differences between arterial beds is important, especially when considering the different physiological role of conduit and resistance arteries (22, 41). In conduit arteries, reduced endothelial NO release in response to stimuli is a central component in the pathophysiology of macrovascular endothelial dysfunction, while NO at the level of microcirculation regulates vascular resistance and match metabolic demands with blood flow. Moreover, FMD has been shown to be more susceptible to traditional risk factors such as age and hypertension, while more distal/peripheral parameters such as RHI appeared to be more sensitive to metabolic risk factors such as body mass index and diabetes mellitus (42). Also, the previous results from the GHS have shown that RHI and RI may be more sensitive to current smoking than FMD (18). However, as these studies primarily aimed to investigate cross-sectional correlates of different vascular function measurement methods without providing comprehensive assessment of smoking exposure as well as sufficient adjustment for confounders, they are less suitable for addressing these issues. More importantly, endothelial dysfunction of resistance, but not conduit arteries has been demonstrated to be associated with increased C-reactive protein and E-selectin levels after adjustment for cardiovascular risk factors in a large cohort of elderly subjects (43). Interestingly, both circulating biomarkers are well-known to be key factors in inflammation-induced atherosclerosis and are highly sensitive to the smoking exposure. Moreover, we recently demonstrated on basis of GHS data that endothelial dysfunction of resistance, but not conduit arteries was associated with increased risk of incident type 2 diabetes mellitus, a well-known consequence of cigarette smoking (21).

Micro- and macrovascular endothelial dysfunction may also indicate different stages of vascular disease as previous data suggested that structural and functional microvascular alterations interact within the vascular continuum of larger arteries, leading to upstream macrovascular endothelial dysfunction over time and thus initiation and progression of atherosclerosis [micro-macro-interaction (44–46)]. Therefore, one might speculate that endothelial dysfunction of resistance artery might represent a risk indicator in the earlier course of disease development, whereas conduit artery endothelial dysfunction may play a more crucial role in patients with established atherosclerosis or manifest CVD (47). However, both resistance as well as conduit arteries may be susceptible to functional alterations due to cigarette smoking as various landmark studies with a focus on conduit arteries could demonstrate (40, 48, 49). Assuming possible differential regulation of endothelial function of conduit and resistance arteries, a deeper insight on the role of smoking exposure, as an important determinant of endothelial dysfunction, on vasculature is clearly needed.

Due to simultaneous assessment of endothelial function of conduit and resistance arteries, the present study represents the largest study so far to directly compare the effects of smoking on endothelial function in different vascular beds in the general population. Within the present analysis, increasing smoking exposure has been clearly related to RHI and RI, suggesting the prominence/stronger influence of exposure for microvascular resistance artery function. In general, smoking exposure was associated with impaired resistance artery endothelial function, whereas smoking cessation among former smokers was related to improved resistance artery endothelial function, reaffirming the cardiovascular benefit of smoking cessation demonstrated by others (50–52). These associations were shown to be less dose-dependent, but rather to reach a point where increasing exposure and prolonged cessation are not accompanied by further impairment or improvement. This observed pattern goes along with previous studies showing that smoking-attributable risk does not follow a monotone dose-dependent course (51, 53). Moreover, these associations seen in the sex- and age-adjusted models were only marginally influenced by further adjustment for a comprehensive set of risk factors and thus it is likely that smoking-induced dysregulated resistance artery endothelial function constitutes an independent risk setting by displaying relevant early vascular damage. Our findings are in good accordance with previous observations that cigarette smoking potentiates endothelial dysfunction of forearm resistance vessels of patients with hypercholesterolemia (35), this endothelial dysfunction can be corrected by infusion of the antioxidant vitamin C (36) as well as by the redox-sensitive eNOS cofactor tetrahydrobiopterin in chronic smokers (37) and patients with atherosclerosis in coronary resistance vessels (54).

In contrast, no association of smoking exposure with FMD, suggestive of conduit artery endothelial function, was observed as FMD may be more sensitive to atherosclerotic progression over time due to preceding microvascular alterations. In this context, microcirculatory alterations in the early course of disease might be still reversible trough adaption, whereas macrocirculatory changes may indicate more progressed disease manifestation trough maladaptation (47). In addition, aspects of artery structure appeared to be influenced by smoking exposure as baseline brachial artery diameter decreased and baseline pulse amplitude increased with increasing smoking exposure. The associations found for the structural components of endothelial function markers were, however, less consistent, which may support previous findings showing that functional impairment precedes structural damage that is present in the later course of disease manifestation (47).

### Strengths and Limitations of the Present Study

The strength of our study includes the possibility to directly compare the role of smoking exposure for the characterization of endothelial dysfunction of conduit and resistance arteries after adjustment for a broad range of confounding variables. Moreover, the large sample size of the population-based GHS across a wide age spectrum and the high-quality data collection through rigorous quality-control procedures throughout the study are notable. However, there were some limitations to our study that need to be mentioned. No analytical and objective read-out of smoking exposure such as the nicotine degradation product cotinine was measured in addition to the rather subjective interview-based exposure assessment that largely depends on the individual compliance. Although we here did not provide a direct proof of oxidative stress and inflammation as major triggers of endothelial dysfunction, previous studies have repeatedly shown that both are central players in smoking-induced endothelial dysfunction [or review see (29, 30)]. Endothelial function was solely measured by non-invasive techniques and we did not include other serum/plasma biomarkers of vascular physiology. As pointed out in a previous review (39), endothelial function measurements may have no significant prognostic value in cohort studies of mostly healthy subjects (55), is not an independent predictor of cardiovascular events in individuals with intermediate cardiovascular risk (56), and, according to the GHS, is unlikely to improve the prognostic value of the European Society of Cardiology risk score (19). Therefore, assessment of several complementary methods for endothelial function determination in different vascular beds as well as measurement of arterial stiffness (e.g., by wall thickness or intima/media ratio), which was shown to improve the risk prediction of cardiovascular events when added to a standard risk factor model (57), may be recommended for better risk stratification. The observational cross-sectional nature of the study does not allow for causal inferences and residual confounding cannot be fully excluded. Also, methodological aspects of endothelial function measurements may have influenced the results since markers derived from digital peripheral arterial tonometry are known to be less NO-mediated and influenced by non-endothelial factors, whereas determination of FMD is based on a less standardized and reliable approach (22).

### Conclusions

In conclusion, we found that cigarette smoking exposure and cessation is associated with an impairment and improvement, respectively, of endothelial function of resistance, but not conduit arteries. These results may provide further mechanistic insight by which smoking initiates cardiovascular dysfunction over time, especially since endothelial dysfunction of resistance vessels will ultimately lead to higher blood pressure. Based on the previous literature, smoking-induced oxidative stress may represent a major contributor to the observed adverse effects on endothelial function and cardiovascular risk (35–37). Further studies are warranted to evaluate underlying pathways for the relationship between smoking exposure and endothelial dysfunction of conduit and resistance arteries.

## Data Availability Statement

The datasets presented in this article are not readily available because the analysis presents clinical data of a large-scale population-based cohort with ongoing follow-up examinations. This project constitutes a major scientific effort with high methodological standards and detailed guidelines for analysis and publication to ensure scientific analyses on the highest level. Therefore, data are not made available for the scientific community outside the established and controlled workflows and algorithms. To meet the general idea of verification and reproducibility of scientific findings, we offer access to data at the local database in accordance with the ethics vote on request at any time. The GHS steering committee, which comprises a member of each involved department and the head of the GHS, convenes once a month. The steering committee decides on internal and external access of researchers and use of the data and biomaterials based on a research proposal to be supplied by the researcher. Requests to access the datasets should be directed to philipp.wild@unimedizin-mainz.de.

## Ethics Statement

The studies involving human participants were reviewed and approved by Ethics committee of the Statutory Physician Board of the State Rhineland-Palatinate [reference number 837.020.07(5555)]. The patients/participants provided their written informed consent to participate in this study.

## Author Contributions

OH, NA, JP, PW, and TM conceived and designed research. OH and NA carried out experiments. OH and AS performed data analysis. OH, NA, AD, and TM drafted the manuscript. MP-N, KL, NP, MM, MB, and JK made critical contribution to the discussion and revised the manuscript. All authors read and approved the final manuscript.

## Conflict of Interest

TM and PW are PI's of the DZHK, Partner Site Rhine-Main, Mainz, Germany. PW and JP are funded by the Federal Ministry of Education and Research (BMBF 01EO1503). The remaining authors declare that the research was conducted in the absence of any commercial or financial relationships that could be construed as a potential conflict of interest.
